# Population pharmacokinetics of a three-day chloroquine treatment in patients with *Plasmodium vivax* infection on the Thai-Myanmar border

**DOI:** 10.1186/s12936-016-1181-1

**Published:** 2016-02-29

**Authors:** Richard Höglund, Younis Moussavi, Ronnatrai Ruengweerayut, Anurak Cheomung, Angela Äbelö, Kesara Na-Bangchang

**Affiliations:** Mahidol Oxford Tropical Medicine Research Unit (MORU), Mahidol University, Bangkok, Thailand; Unit for Pharmacokinetics and Drug Metabolism, Department Pharmacology, Sahlgrenska Academy at the University of Gothenburg, Gothenburg, Sweden; Mae Sot General Hospital, Mae Sot, Tak province Thailand; Center of Excellence for Molecular Biology and Pharmacology of Malaria and Cholangiocarcinoma, International College of Medicine, Thammasat University, Bangkok, Pathumtanee Thailand

**Keywords:** *Plasmodium vivax*, Chloroquine, Desethylchloroquine, Population pharmacokinetics, Drug resistance

## Abstract

**Background:**

A three-day course of chloroquine remains a standard treatment of *Plasmodium vivax* infection in Thailand with satisfactory clinical efficacy and tolerability although a continuous decline in in vitro parasite sensitivity has been reported. Information on the pharmacokinetics of chloroquine and its active metabolite desethylchloroquine are required for optimization of treatment to attain therapeutic exposure and thus prevent drug resistance development.

**Methods:**

The study was conducted at Mae Tao Clinic for migrant worker, Tak province, Thailand. Blood samples were collected from a total of 75 (8 Thais and 67 Burmeses; 36 males and 39 females; aged 17–52 years) patients with mono-infection with *P. vivax* malaria [median (95 % CI) admission parasitaemia 4898 (1206–29,480)/µL] following treatment with a three-day course of chloroquine (25 mg/kg body weight chloroquine phosphate over 3 days). Whole blood concentrations of chloroquine and desethylchloroquine were measured using high performance liquid chromatography with UV detection. Concentration–time profiles of both compounds were analysed using a population-based pharmacokinetic approach.

**Results:**

All patients showed satisfactory response to standard treatment with a three-day course of chloroquine with 100 % cure rate within the follow-up period of 42 days. Neither recurrence of *P. vivax* parasitaemia nor appearance of *P. falciparum* occurred. A total of 1045 observations from 75 participants were included in the pharmacokinetic analysis. Chloroquine disposition was most adequately described by the two-compartment model with one transit compartment absorption model into the central compartment and a first-order transformation of chloroquine into desethylchloroquine with an additional peripheral compartment added to desethylchloroquine. First-order elimination from the central compartment of chloroquine and desethylchloroquine was assumed. The model exhibited a strong predictive ability and the pharmacokinetic parameters were estimated with adequate precision.

**Conclusion:**

The developed population-based pharmacokinetic model could be applied for future prediction of optimal dosage regimen of chloroquine in patients with *P. vivax* infection.

## Background

Malaria remains one of the major global public health problems in the tropics and subtropics including Southeast Asia. The most recent World Malaria Report revealed an estimated 3.3 billion people at risk, 198 million estimated cases, and 584,000 deaths, of which 90 % occurred in Africa [1]. Apart from drug resistance in *Plasmodium falciparum*, the “sleeping giant” in the Greater Mekong subregion is *P. vivax* malaria, which has now become resistant to the blood schizontocide chloroquine in some of the Southeast Asian countries, notably Indonesia [2, 3]. Chloroquine resistance is linked to increasing rates of anaemia and may be an important factor in severe *P. vivax* malaria [3]. The burden of *P. vivax* varies widely with the World Health Organization (WHO) estimating it being responsible for approximately 12–22 million cases worldwide annually [1]. The disease is rarely life-threatening, but morbidity from a prolonged illness and the possibility of relapses from a persistent hepatic form (hypnozoite) which occurs more frequently with the tropical form of *P. vivax* found in Southeast Asian countries, is of major concern and cause considerable economic loss.

In Thailand, chloroquine and the tissue schizontocide primaquine have remained the mainstay treatment of *P. vivax* infection for more than 60 years with a conserved clinical efficacy of virtually 100 % [4–7]. To date, there has been no clinico-parasitological evidence of chloroquine resistant *P. vivax* in Thailand, although a trend in gradual decline of in vitro sensitivity to the drug has been documented in some areas of the country, particularly along the Thai-Myanmar border [8, 9]. It is possible that resistant levels may remain obviously below the threshold of detectability by the in vivo assessment. The accumulating reports of chloroquine resistant *P. vivax* in other parts of the world during the past three decades particularly in Southeast Asian region such as Indonesia [10], Papua New Guinea [11–14], Irian Jaya [15–18], Myanmar [19–21] and Vietnam [22], emphasize the need for closely and continuously monitoring clinical efficacy in conjunction with in vitro sensitivity with confirmed adequacy of anti-malarial systemic drug exposure [23]. The information obtained would facilitate the early recognition of treatment failures and adjustment of treatment policy. Optimization of chloroquine treatment is essential to attain therapeutic exposure and thus prevent resistance development to the drug. Inadequate drug exposure may lead to subtherapeutic concentrations of chloroquine and an increased risk of severe vivax malaria as well as the development of resistant strains of *P. vivax.* The aim of the study was to investigate the pharmacokinetics of chloroquine and its active metabolite desethylchloroquine following treatment with a three-day standard course of chloroquine in patients with *P. vivax* infection on the Thai-Myanmar border.

## Methods

### Patients and study design

The study was conducted at Mae Tao clinic for migrant workers, Tak Province, Thailand. Prior to study, approval of the study protocol was obtained from the Ethics Committee of the Ministry of Public Health of Thailand. The study was part of the clinical study conducted during 2010–2011 to monitor the clinical efficacy and in vitro sensitivity of *P. vivax* isolates to chloroquine in an area along the Thai-Myanmar border [7]. Written informed consents were obtained from all patients before study participation. A total of 75 (8 Thai and 67 Burmese; 36 males and 39 females; aged 17–52 years) patients with *P. vivax* mono-infection [median (95 % CI) admission parasitaemia 4898 (1206–29,480)/µL] were included in the study [7]. In brief, patients were treated with the standard three-day chloroquine (Government Pharmaceutical Organization of Thailand, 250 mg chloroquine phosphate *per* tablet) regimen given at a total dose of 25 mg base/kg body weight over 3 days (10 and 5 mg/kg at 0 and 6–12 h on day 0, and 5 mg/kg each on day 1 and day 2) and primaquine (Government Pharmaceutical Organization of Thailand, 15 mg base per tablet) given at daily doses of 15 mg base/kg body weight daily for 14 days starting from the second day (day 1) of chloroquine treatment. Chloroquine and primaquine dose administration during the first 3 days (days 0, 1 and 2) were administered with a glass of 250 mL drinking water under the supervision of a medical staff. Patients were closely observed for at least 30 min after drug ingestion.

All patients were admitted to the clinic during the course of treatment or until signs and symptoms of malaria disappeared. Prior to treatment, a blood sample (5 mL) was collected from each patient for in vitro sensitivity testing of *P. vivax* isolates to chloroquine and determination of baseline anti-malarial drug concentrations (chloroquine and its active plasma metabolite desethylchloroquine). Patients were requested to return for follow-up on days 7, 14, 21, 28, 35 and 42, or at any time if fever or symptoms suggestive of malaria developed. At each visit, a parasite count was performed (Giemsa stain), and a detailed questionnaire for general symptoms was recorded. Malaria blood smears were obtained on enrollment and thereafter, twice daily until two consecutive slides were confirmed to be negative, as well as at every follow-up visit. Thick films were screened for 200 oil-immersion fields before declaring a slide negative. Asexual parasites and gametocytes were separately counted against 200 white blood cells (WBCs); if the parasite density was too numerous to count on the thick film, the number of parasites per 2000 red blood cells (RBCs) on the thin film were counted. Clinical efficacy of the three-day course of chloroquine was evaluated in the group of patients who completed the 42-day follow-up period. The classification of the therapeutic outcome was according to the WHO protocol [23].

### Blood sampling and drug analysis

Blood samples were collected at specified time points, *i.e.,* pre-dose and at 1, 6, 12, 24, 25, 36, 48 and 49 h after the first dose for measurement of chloroquine and desethylchloroquine concentrations. Blood samples were also collected during the follow-up period at day 7, 14, 21, 28, 35 and 42 after the initiation of the treatment. Concentrations of chloroquine and desethylchloroquine in plasma samples were measured using high performance liquid chromatography according to the method of Cheomung and colleagues [24]. The lower limit of quantification (LOQ) of the assay was 2 ng/mL for both chloroquine and desethylchloroquine. The assay accuracy (expressed as % relative error: % RE) of the quality control samples used during the sample analysis for both chloroquine and desethylchloroquine ranged from 0.25 to 5.7 %. The assay precision (expressed as coefficient of variation: %CV) were less than 5 % for both chloroquine and desethylchloroquine.

### Population pharmacokinetics

#### Modelling and data handling

Concentration–time data of chloroquine and desethylchloroquine, transformed into their natural logarithms, were analysed using the mixed-effects modelling in NONMEM^®^ (version 7.12; ICOM Development Solutions, Ellicot City, MD, USA) and the output results and graphical plots were handled using the statistical analysis programs R (version 2.15.1; Free Software Foundation, Boston, MA, USA) and R-package Xpose (version 4.3.5; Uppsala University, Uppsala, Sweden). The observations that were below the limit of quantification were excluded from the pharmacokinetic analysis. The first-order conditional estimation (FOCE) method was used throughout the modeling. Model evaluation was based on visual inspection of diagnostic plots, precision of parameters and the objective function value (OFV; proportional to −2 Log likelihood) [25]. For nested models, the difference in OFV is approximately Chi squared distributed and it can therefore be used in model discrimination.

For a one parameter difference between models, 3.84 correspond to a *p* value of 0.05. Population pharmacokinetic models were constructed to evaluate the concentration–time data for chloroquine and desethylchloroquine and to identify any covariates that could describe between subject variability (BSV). A metabolite model was implemented to describe the pharmacokinetics of chloroquine and desethylchloroquine. One, two-and three compartment models were initially investigated both for the parent drug and metabolite. Different models for the elimination of chloroquine and its metabolite were evaluated. A first-order absorption model and a transit compartment absorption model with 1–10 transit compartments were investigated to describe the absorption of chloroquine. Relative bioavailability was added with a typical value of 100 % with an estimate of between-subject variability. Between-subject variability was added exponentially, resulting in log-normal distributed parameters:

$${\text{P}}_{\text{i}} = {\text{P}}_{\text{p}} {\text{e}}^{{{\upeta}_{\text{i}} }}$$

where P_i_ is the true value of the parameter for the individual and P_p_ is the typical or population value of the parameter. P_p_ is the fixed effect parameter estimated from the structural model and $\upeta_{\text{i}}$ represents the difference between P_i_ and P_p_.

An additive residual variability (RUV) model was applied according to:

$${\text{C}}_{\text{obs}} = {\text{C}}_{\text{p}} + \varepsilon_{\text{ad}}$$

where C_obs_ is the observed drug or metabolite concentration and C_p_ is the concentration predicted by the model and ε_ad_ represents the difference between these values. An additive model on log-transformed data is equivalent to an exponential model.

The most adequate structural model with random effects (base model) was further developed to include covariates using a stepwise forward addition (*p* = 0.05) of covariates, followed by a stepwise backward elimination procedure (*p* = 0.001). Relationships between all parameters estimated in the base model and covariates*, i.e.,* body weight (BW), age, sex, parasite clearance time (PCT) and fever clearance time (FCT) were evaluated. The covariate was retained in the final model if its removal resulted in an increase in the objective function of ≥10.83 points (*p* < 0.001) from the full model. BW was applied as a covariate on all CL and V values as a power model according to equation:

$${\text{P}}_{{\text{t}}} = {{\uptheta }}_{1} \cdot \left( {\frac{{{\text{BW}}}}{{{\text{median BW}}}}} \right)^{{{{\uptheta }}_{2} }}$$

where P_t_ is the typical population value of the parameter for the population; θ_1_ represents the estimate of P in an individual with median BW; and θ_2_ is the fractional change in P_t_ with each kilogram change in BW from median BW. BW was allometrically scaled and θ_2_ was defined as 0.75 and 1 when applied on CL and V, respectively.

The covariate model for continuous covariates such as FCT was exemplified by the following equation:

$${\text{VP}}_{{\text{t}}} = {{\uptheta }}_{1} \cdot \left[ {1 \,+\, {{\uptheta }}_{2} {\mkern 1mu} \,\times\, {\mkern 1mu} ({\text{FCT}} \,-\, {\text{median FCT}})} \right]$$

where P_t_ is the typical value of parameter P; θ_1_ represents the estimate of P in an individual with median FCT; and θ_2_ the fractional change in P with each change in unit of FCT from median FCT.

All clearance and distribution parameters are reported as the ratio of the parameter and bioavailability since oral dosing was not accompanied by an intravenous dose. The pharmacokinetic population parameters estimated from the final covariate model were used to calculate terminal half-life for chloroquine and desethylchloroquine.

Bootstrap diagnostics were performed using 1000 re-sampled datasets. The precision was described as a relative standard error. A visual predictive check (VPC) is a tool for the evaluation of the predictive ability and the appropriateness of a model and was done by performing simulations of 1000 observations at each time point for the real observations in the data set with the final covariate model. The median and 95 % prediction intervals of the simulated data and the true observations were plotted against time. The predictive ability of the model was assumed to be adequate if less than 10 % of the observed concentrations fell outside the prediction interval.

## Results

A total of 75 patients with *P. vivax* malaria were included in the analysis. All had completed a 42 days follow-up period. All patients showed good response following treatment with no reappearance of parasitaemia. The treatment was well-tolerated. All patients showed satisfactory response to treatment with 100 % cure rate within the follow-up period of 42 days. Median (95 % CI) parasite clearance time (PCT: the time taken for the parasite count to fall below the level of microscopic detection) and fever clearance time (FCT: the time taken for the temperature to return to normal*, i.e.,* <37.3 °C) were 30 (18–36) and 24 (12–42) hours, respectively. Neither recurrence of *P. vivax* parasitaemia nor appearance of *P. falciparum* occurred.

### Population pharmacokinetic models

**Fig. 1:**
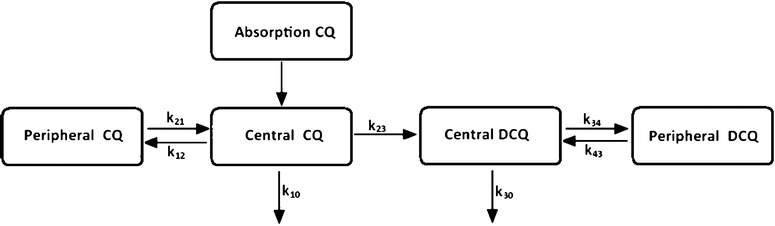
A two-compartment model (central and peripheral) with a one transit compartment model for the absorption of chloroquine into the central compartment and a first-order transformation of chloroquine into desethylchloroquine with an additional peripheral compartment added to desethylchloroquine. *CQ* is chloroquine compartments and *DCQ* represents desethylchloroquine compartments. *k* represents the rate constant between different compartments

The final data set included in the pharmacokinetic modeling consisted of 1405 observations of both chloroquine and desethylchloroquine from 75 individuals (less than 5 % of the samples below the lower limit of quantification). The final model for chloroquine and desethylchloroquine following a three-day chloroquine dose regimen was a two-compartment model for both chloroquine and its metabolite (*p* < 0.01) with a one transit compartment model for the absorption of chloroquine (Fig. 1). The parameter describing the transformation of chloroquine into desethylchloroquine (CL_m_) was fixed to 18 % of the transformation clearance from parent drug to metabolite [26]. One transit compartment described the absorption phase adequately and the relative bioavailability were retained in the final model (*p* < 0.05). In the final model, BSV was kept on the apparent volume of distribution of desethylchloroquine, relative bioavailability and the apparent volume of distribution in the peripheral compartment of chloroquine and desethylchloroquine. Adding between-subject variability on the mean transit time resulted in high relative standard errors on this parameter (149 %, based on 59 successful bootstraps runs out of 100) and was not kept in the model.

**Fig. 2:**
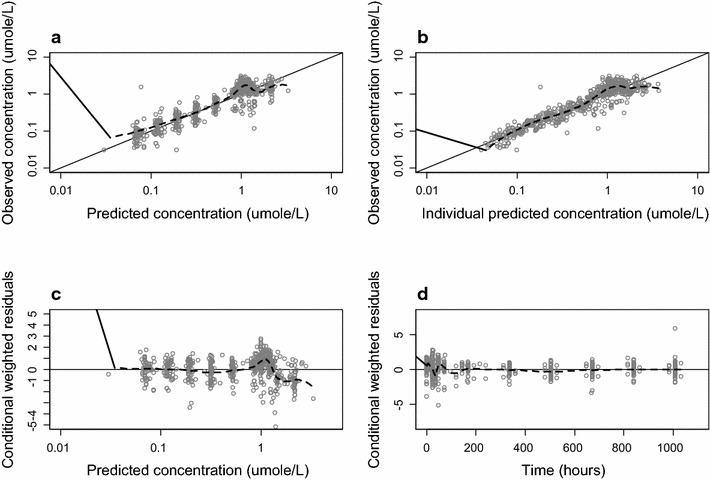
Goodness of fit plots of chloroquine. Plots of the observed versus population predicted concentrations (**a**) and observed versus individual predicted concentrations (**b**). Weighed individual residuals versus individual predictions (**c**) and weighed residuals versus time (**d**). The *black line* is a non-parametric smoother describing the trend and the *black line* is the line of unity

**Fig. 3:**
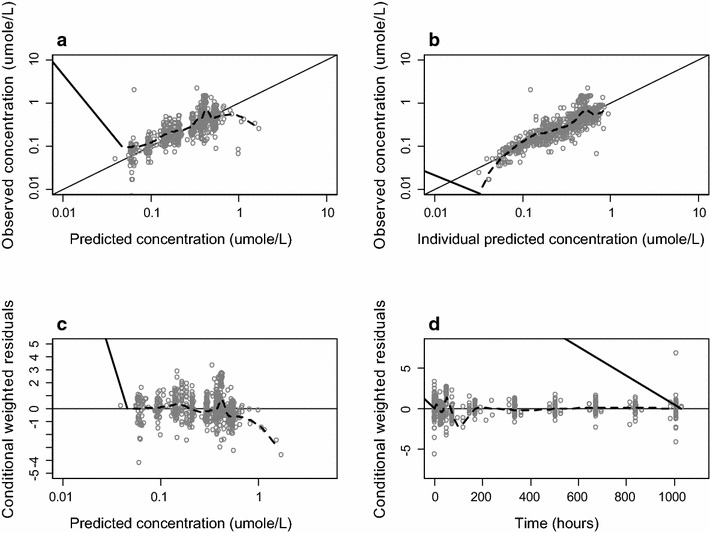
Goodness of fit plots of desethylchloroquine. Plots of the observed versus population predicted concentrations (**a**) and observed versus individual predicted concentrations (**b**). Weighed individual residuals versus individual predictions (**c**) and weighed residuals versus time (**d**). The *black line* is a non-parametric smoother describing the trend and the *black line* is the line of unity

**Fig. 4:**
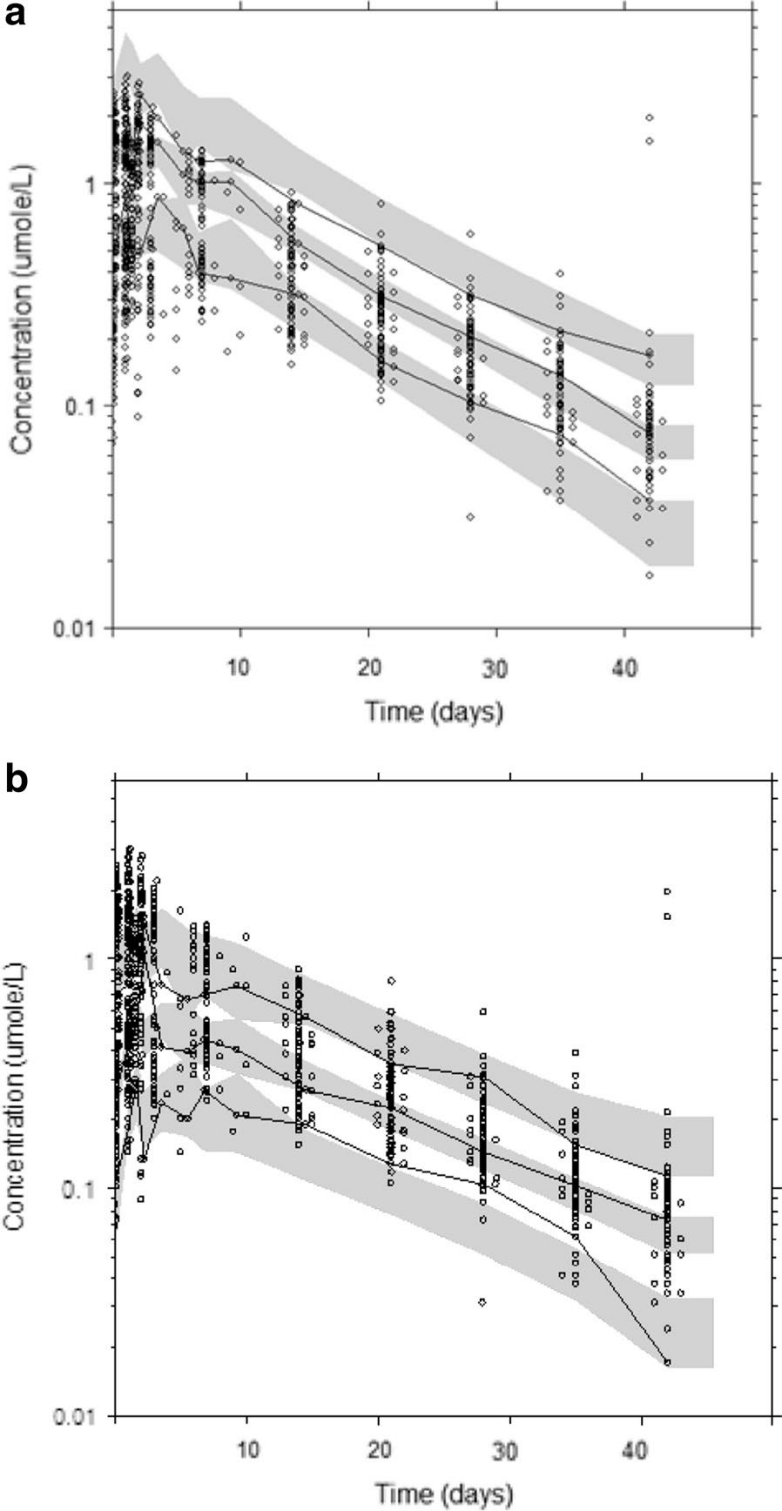
Plots from the visual predictive check for chloroquine (**a**) and desethylchloroquine (**b**) observations. The *middle solid lines* represent the median of simulated predictions by the final model. The *dashed black lines* represent the corresponding percentiles for the true observations. The *black dots* are the true observations and the *grey shaded* areas are the 95 % confidence intervals for the simulations. The decline in the *upper* percentiles of desethylchloroquine is due to base line values in the subjects

No covariates were added in the final model. FCT was a significant covariate for V_P CQ_/F in the forward step but was not retained in the backward step. The basic goodness of fit plots exhibited adequate description of the data (Fig. 2). Parameter estimates and their precision for the final model and estimates from the non-parametric bootstrap are listed in Table 1. The calculated half-lives are presented in Table 1. The visual predictive check indicated a strong predictive ability of the model for the dataset where less than 10 % of the observations were outside the 95 % prediction interval (Figs. 3, 4).

**Table 1:** Objection function values, parameter estimates and their precision for the final covariate model and the bootstrap. The bootstrap estimates are derived from the final covariate model

Parameter	Estimated from	
	Final covariate model (RSE)	Bootstrap 95 % CI
Pharmacokinetic parameter^a^		
MTT (h)	0.773 (43.1)	0.809–2.38
CL_CQ_/F (L/h)	6.13 (3.40)	5.74–6.55
V_C CQ_/F (L)	468 (16.0)	137–529
V_P CQ_/F (L)	1600 (5.21)	1470–1800
Q_CQ_/F (L/h)	37.7 (18.9)	31.1–69.0
t_1/2_ CQ (days)	10.7	
CL_DCQ_/F (L/h)	2.04 (3.50)	1.90–2.18
V_C DCQ_/F (L)	2.27 (14.1)	1.62–2.90
V_P DCQ_/F (L)	566,257 (14.4)	198–341
Q_DCQ_/F (L/h)	31.46 (12.3)	1.11–1.83
t_1/2_ DCQ (days)	8.74	
Interindividual variability^b^		
BSV V_C DCQ_	48.7 (47.5)	17.9–71.1
BSV V_P CQ_	20.0 (61.6)	8.25–31.9
BSV V_P DCQ_	86.8 (30.5)	49.1–116
BSV F	19.4 (31.7)	13.1–25.4
Residual variability^c^		
Proportional error CQ	0.401 (5.34)	0.360–0.444
Proporional error DCQ	0.431 (4.97)	0.393–0.479

*MTT* mean transit time of the absorption, *CLCQ/F* apparent clearance of CQ for transformation into desethylchloroquine, *VC CQ/F* apparent volume of distribution for CQ central compartment, *VP CQ/F* apparent volume of distribution for CQ peripheral compartment, *QCQ/F* apparent intercompartmental clearance for CQ, *t*_*1/2*_ *CQ* half-life of chloroquine, *KFCT* constant describing the fraction of change in peripheral volume of distribution of chloroquine with each unit of fever clearance time, *CL DCQ/F* apparent clearance of DCQ, *VC DCQ/F* apparent volume of distribution for DCQ central compartment, *VP DCQ/F* apparent volume of distribution of DCQ peripheral compartment, *QDCQ/F* apparent intercompartmental clearance for DCQ, *t*_*1/2*_ half-life of desethylchloroquine. *BSV* is the between subject variability

^a^Listed as estimates and their relative standard errors (RSE; %) in parenthesis. RSE is calculated based 603 succesfull bootstrap runa (out of 1000) according to: 100 × Standard deviation/Average, 95 % CI is the 95 % confidence interval of the bootstrap parameter estimates

^b^Listed as coefficient of variation (CV; %) and their RSE (%) in parenthesis

^c^Listed as CV (%) and their RSE (%) in parenthesis

## Discussion

Chloroquine remains the anti-malarial drug which is widely used in the tropics due to its safety, availability and low cost. The drug has now been rendered completely ineffective for treatment and prophylaxis of *P. falciparum*, but for *P. vivax*, *P. ovale* and *P. malariae,* it is still in use. For optimization of dosage regimen of chloroquine for the treatment or prophylaxis of these infections, plasma/blood drug concentrations and pharmacokinetic analysis are necessary. Nevertheless, the pharmacokinetics of chloroquine is not well understood. Previous studies involved small number of subjects and in some cases, with limitation of sensitivity of analytical methods and pharmacokinetic modeling techniques [27–32]. In addition, a wide range of inter-individual variability in the estimated pharmacokinetic parameters hurdles optimization of dose regimen of chloroquine for both clinical applications particularly in patients infected with *P. vivax*.

The large variability between individuals in the pharmacokinetic parameters of chloroquine makes population approaches a convenient method to assess the pharmacokinetic characteristics of the drug. Inclusion of concentration data of the active metabolite desethylchloroquine in the analysis is of further relevance as this metabolite has been shown to exhibit significant anti-malarial activity [33]. The final covariate model included a two-compartmental disposition for both chloroquine and desethylchloroquine with an adequate accuracy in the estimated parameters. The multi-exponential declines for both parent compound and metabolite are in consistency with previous reports [34–37]. For chloroquine, the estimates of absorption rate constant, the total apparent volume of distribution, and elimination half-life of chloroquine are in agreement with previously reported values. The apparent elimination clearance is lower compared to previous studies. This is probably due to the use of different sample matrixes [26–41]. Estimates of desethylchloroquine parameters such as apparent elimination clearance, apparent total volume of distribution and elimination half-life were relatively lower than reported values [26, 34–39]. The fact that reports of desethylchloroquine parameter estimations have been limited, might offer a skewed distribution of the values of the parameters. The underestimation of the parameters could also be due to the fixation of CL_m_ to 18 %, an estimation based on the fraction desethylchloroquine of the total chloroquine dose recovered in urine. In the study reported by Karunajewa et al. [35], the same approximation of CL_m_ was used and might also have underestimated their obtained pharmacokinetic parameters of desethylchloroquine. Besides desethylchloroquine, bidesethylchloroquine is produced by secondary metabolism of desethylchloroquine [42] and the formation of the third metabolite might also be a result of further metabolism of bidesethylchloroquine. This suggests that the amount of chloroquine transformed into desethylchloroquine should be assessed by estimating the amount of all secondary metabolites and including them in the estimation of the fraction desethylchloroquine formed. As chloroquine is metabolized up to 30–50 % by the liver and the mainly formed metabolites are (mono) desethylchloroquine and bidesethylchloroquine, the fraction of desethylchloroquine formed needs to be reassessed.

The quantification of a relationship between the drug concentration and response (pharmacokinetic-pharmacodynamic relationship) enables the identification of drug target levels. Biomarkers of response for the treatment of malaria have been collected and since the target of action of chloroquine is in infected red blood cells, it would most probably be uncomplicated and straightforward to characterize the relationship between blood concentrations and parasitaemia. The concentrations of chloroquine or desethylchloroquine associated with adequate treatment of *P. vivax* malaria have not been established rigorously. The minimum effective concentration (MEC) of chloroquine in plasma or serum of 15–30 ng/mL or in whole blood of 90 ng/mL have been suggested [45, 46]. Furthermore, patients with parasitaemia in the presence of chloroquine and desethylchloroquine concentration in whole blood of greater than 100 ng/mL is considered chloroquine resistant [16, 47, 48]. Unfortunately, this threshold level could not be determined in this group of patients as none had treatment failure following this standard regimen of chloroquine. In a recent study conducted in Bolivia, South America, chloroquine resistance *P. vivax* was reported in 6.5 % patients [49]. Chloroquine and desethylchloroquine in whole blood on day 7 and the day of parasite recrudescence in ten patients were 197–535 and 75–223 ng/mL, respectively. Six out of these ten patients has drug concentrations above the MEC.

## Conclusion

A population pharmacokinetic model for chloroquine incorporating desethylchloroquine has been developed and validated with an adequate precision on the parameters. The determination of a more realistic fraction of desethylchloroquine formed would also be needed for future studies. This would create the possibility of more optimal exposure prediction for both compounds and optimization of malaria treatment in P.*vivax* monoinfection. Prompt and effective treatment would lead to efficacious killing of the malaria parasites and the prevention of resistance development to chloroquine.
